# Unveiling the wonders of bacteria-derived extracellular vesicles: From fundamental functions to beneficial applications

**DOI:** 10.1016/j.heliyon.2025.e42509

**Published:** 2025-02-06

**Authors:** Mariam Rima, Mariam Dakramanji, Elie El Hayek, Tia El Khoury, Ziad Fajloun, Mohamad Rima

**Affiliations:** a Laboratory of Applied Biotechnology (LBA3B), Azm Center for Research in Biotechnology and Its Applications, EDST, Lebanese University, 1300, Tripoli, Lebanon; b Department of Biological Sciences, Lebanese American University, P.O. Box 36, Byblos, Lebanon; c Department of Biology, Faculty of Sciences 3, Campus Michel Slayman Ras Maska, Lebanese University, 1352, Tripoli, Lebanon

**Keywords:** Extracellular vesicles, Biofilm, Antimicrobial resistance, Drug delivery

## Abstract

Extracellular vesicles (EVs), are critical mediators of intercellular communication and exhibit significant potential across various biomedical domains. These nano-sized, membrane-encapsulated entities have captured substantial interest due to their diverse roles in pathogenesis and promising therapeutic applications. EVs manage numerous physiological processes by transferring bioactive molecules, including proteins, lipids, and nucleic acids, between cells. This review delves into the factors influencing the properties of EVs, such as temperature and stress conditions, which collectively influence their size, composition, and functional attributes. We also describe the emerging roles of EVs, emphasizing their involvement in microbial interactions, immune modulation, antimicrobial resistance spread and their potential as innovative diagnostic and therapeutic instruments. Despite their promising applications, the advancement of EV-based therapies faces several challenges, which will also be discussed. By elucidating these critical elements, we aim to provide a comprehensive overview of the transformative potential of EVs in revolutionizing diagnostics and therapeutics in medicine.

## 1 Extracellular vesicles: classification, isolation, composition and functions

The secretion of cellular components across the plasma membrane constitutes an essential intrinsic process to all life forms, facilitating the interaction of organisms with their respective environments. One mechanism by which cells execute this function is through the release of nano-sized vesicles, known as extracellular vesicles (EVs) [1]. These spherical nanoscale structures are derived from the lipid membranes of the cell surface and secreted by all domains of life including both prokaryotes and eukaryotes [2,3]. EVs can be classified based on their origin, size, biogenesis, and release pathways. In Eukaryotes, three EVs types are distinguished: (i) ***Microvesicles*** ranging from 200 nm to 1 μm, formed by an outward budding from the plasma membrane; (ii) ***Exosomes*** *ranging from 30 to* 150 nm*,* produced by the fusion between multivesicular bodies and the plasma membrane; (iii) ***Vesicular apoptotic bodies*** ranging from 1000 nm to 5000 nm, generated during programmed cell death (Table 1) [[4], [5], [6]]. Among these three main types of vesicles produced by eukaryotic cells, ***Cell derived Microparticles*** and ***microvesicles*** are often used interchangeably in the literature, despite subtle differences in their size, formation process, content, and functional roles. Microparticles typically range from 100 to 1000 nm and are formed through membrane budding in response to cell activation or stress; Microvesicles, on the other hand, tend to be slightly larger, ranging from 200 to 1000 nm, and are released during normal physiological processes such as immune modulation and cell communication. Both types of vesicles carry similar cargo, including proteins, lipids, RNA, and DNA, reflecting the state of their parent cell. However, microparticles are often linked to pathological processes like thrombosis and inflammation, whereas microvesicles are more associated with routine cellular functions like signaling and tissue repair. Additionally, microparticles often emerge during stress or injury, while microvesicles play a more proactive role in physiological cell communication, reflecting their distinct contributions to intercellular interactions and biological processes [7,8].

**Table 1:** Characteristics of EVs secreted by bacteria and eukaryotic cells.

Source	Type	Size (nm)	Biogenesis	Release Pathway
Eukaryotes	Exosomes	30–150	Inward budding of MVBs	Fusion of MVBs with plasma membrane
	Microvesicles	200–1000	Outward budding of the plasma membrane	Direct shedding
	Apoptotic Bodies	1000–5000	Apoptosis (cell death)	Fragmentation of dying cells
Gram-negative bacteria	Outer membrane vesicles	10–300	Outward budding of the outer membrane	Direct shedding/scission
Gram-positive bacteria	Membranes vesicles	20–400	Budding or shedding from the cell wall or membrane	Direct shedding/scission
Bacteria	Exosome-like Vesicles	<100	Formed from the outer membrane in gram negative bacteria and derived from the plasma membrane, in gram positive bacteria	Pinched off from the membrane and released into the extracellular environment
Bacteria	Apoptotic Bodies	500–1000	Formation of vesicles resembling apoptotic bodies seen in eukaryotic cells	Localized destabilization and blebbing
Bacteria	Protoplast-derived Vesicles	50–150	Localized membrane deformation leads to the formation of spherical blebs, which gradually separate from the protoplast membrane	Blebs pinch off from the plasma membrane, forming stable, free-floating Protoplast derived vesicles

**Fig. 1:**
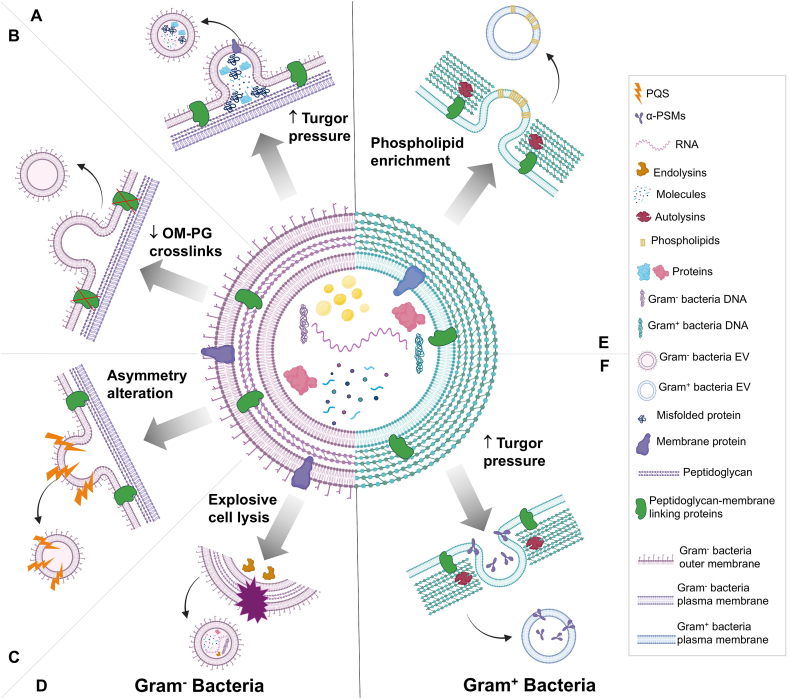
**Mechanistic models of extracellular vesicles biogenesis in Gram-negative and Gram-positive bacteria.** Blebbing and explosive cell lysis are among the main biogenesis mechanisms of Gram-negative bacterial EVs. Blebbing can be initiated by: (A) the buildup of misfolded proteins in the periplasm leading to an increased turgor pressure, (B) the loss of crosslinks between the outer membrane (OM) and peptidoglycan (PG), and (C) the alteration of the membrane composition that could result from *Pseudomonas Quinolone* Signals (PQS) or from a decreased expression of the ABC transport system. The explosive cell lysis mechanism (D) is linked to endolysins that degrade the PG. Gram-positive bacterial EVs are mainly secreted through budding which is induced by phospholipids enrichment (E), or the increased turgor pressure (F) caused by Phenol-soluble-modulins (PSMs) and autolysins that alter the cytoplasmic membrane content and break down the peptidoglycan components respectively.

In recent years, the scientific community has witnessed an escalating interest in the EVs secreted by pathogenic entities, including bacteria. This discovery, dating back over 60 years, has since garnered considerable research attention. Since then, Gram-negative bacteria became the primary focus of research in the field of EVs. Today, they are recognized for secreting three types of EVs namely (i) **outer membrane vesicles** (OMVs); (ii) **outer-inner membrane vesicles** (O-IMVs); and (iii) **explosive outer membrane vesicles** (E-OMVs) [9] (Fig. 1). On the other hand, it was previously believed that the structure of the peptidoglycan cell wall would prevent EV production in Gram-positive bacteria. Nonetheless, recent studies have successfully isolated and identified 20–400 nm spherical lipid bilayer structures in the supernatants of Gram-positive bacteria, so called **membrane vesicles** indicating that EVs are prevalent across the prokaryote domain. Since then, studies have started to explore also the physiology of Gram-positive EVs [[10], [11], [12]]. Recent studies concluded that the formation of Gram-positive bacterial MVs is mainly driven by the expansion of specific lipid-rich areas within the cytoplasmic membrane. This process weakens the thick peptidoglycan layer in the presence of endolysins, aiding in the release of MVs [13]. In addition to those types of vesicles, we distinguish, **Exosome-like Vesicles, Apoptotic Bodies**, and **Protoplast-derived Vesicles** derived also from bacteria [[14], [15], [16], [17]]. These diverse structures share some features with EVs derived from eukaryotes but are distinguishable from them (Table 1). Additionally, subtypes of EVs derived from bacteria differ in their structures, activities, and the immune pathways they activate, making them a fascinating topic of study (Table 2). Notably, in alignment with MeSH terminology, we have opted to use the term "EVs" (Extracellular Vesicles) throughout the manuscript. However, it is important to emphasize that this review focuses exclusively on EVs derived from bacteria.

**Table 2:** Characteristics and functional roles of bacterial extracellular vesicles.

Type of bacterial EV	Structure	Roles and activities	Importance	Immune system signaling pathway	References
Outer membrane vesicles OMVs	Derived from the outer membrane of Gram-negative bacteria; spherical, lipid bilayer vesicles	Horizontal gene transfer, toxin delivery, nutrient acquisition, biofilm formation, immune modulation	Facilitates bacterial survival, pathogenesis, and communication; transfers antibiotic resistance genes	Activates Toll-like receptors (TLRs) via lipopolysaccharides (LPS) or other PAMPs	[[18], [19], [20]]
Membrane vesicles MVs	Produced by Gram-positive bacteria; derived from plasma membranes	Adhesion, virulence factor transport, inter-bacterial communication, immune evasion	Critical for biofilm development and bacterial-host interactions	Stimulates TLRs or other pattern recognition receptors (PRRs)	[21]
Exosome like vesicles	Smaller vesicles (<100 nm); lipid bilayer structure; primarily in Gram-positive bacteria	Inter-bacterial communication, host immune modulation	Emerging role in intracellular communication and immune system interaction	May interact with TLRs to modulate immune responses	[22,23]
Apoptotic bodies	Larger vesicles formed during bacterial cell death; contain DNA, proteins, and cellular debris	Disseminates bacterial antigens, spreads virulence factors	Facilitates survival under stress, triggers immune responses	Activates innate immune pathways via DAMPs recognized by TLRs	[24]
Protoplast derived vesicles	Shed from Gram-positive bacteria after wall degradation; lipid bilayer with internal contents	Interaction with host cells, delivery of bacterial components	Helps bacteria adapt to environmental changes and host defenses	Stimulates immune responses depending on cargo, activating PRRs	[14]

Due to their distinct structures, Gram-positive and Gram-negative bacteria exhibit different pathways for EVs production (Fig. 1). Various models have been proposed to elucidate the diversity and complexity of EV biogenesis in Gram-negative bacteria. One such mechanism suggests that vesiculation in Gram-negative bacteria could be driven by the explosive cell lysis triggered by endolysins, that degrade the bacterial peptidoglycan [25]. The accumulation of misfolded proteins or other fragments within the periplasm was also recognized as a possible route for vesicle formation in Gram-negative bacteria (Fig. 1). This buildup creates a periplasmic turgor pressure on the outer membrane, causing it to bulge, pinch off, and form a vesicle [26]. An association between the outer-membrane peptidoglycan junctions and OMVs biogenesis has been well established in Gram-negative bacteria. Indeed, the loss of these cross-links, involved in maintaining the integrity of the Gram-negative bacteria's envelope, leads to the production of OMVs [27]. Another proposed model involves ABC (ATP-binding cassette) transport system with predicted import role. A decreased expression of this transporter leads to the asymmetrical organization of phospholipids and their accumulation on the outer leaflet of the outer membrane triggering vesiculation in Gram negative bacteria [28,29]. The membrane's composition could also be altered by the introduction of molecules such as *Pseudomonas* Quinolone Signals (PQS), which modify the membrane's curvatures and thus promote the release of OMVs [27]. A similar scenario is observed in Gram-positive bacteria where a lipid distribution within the cytoplasmic membrane is implicated in EVs discharge [30,31]. It is noteworthy that the single membrane and thick peptidoglycan-rich cell wall of Gram-positive bacteria renders vesicles formation more challenging [10,32]. Consequently, specific modulators are involved during EVs biogenesis in Gram-positive bacteria to control membrane fluidity and cell wall permeability [10,33,34]. For instance, *Staphylococcus aureus* EVs require small amphipathic α-helical peptides, alpha-type phenol-soluble modulins to alter the bacterial cytoplasmic membrane, as well as peptidoglycan crosslinking and autolysin activity modulate to modify the integrity of the cell wall [32,35]. By comparing EVs secretion in wild type bacteria within isogenic mutant lineages, numerous studies have demonstrated that a large pool of genes is involved in EVs biogenesis, particularly in Gram-negative bacteria, where more than 150 genes were shown to be involved in the vesiculation process of OMVs. Similarly, even though Gram-positive bacteria have only received scant research attention to date, it is evident that their EVs production is a complex process and does not solely depend on one gene [[35], [36], [37], [38], [39]], rather, it depends on a complex global network including the regulator gene *sigB*. It's worth mentioning, that some genes involved in MVs vesiculogenesis are also intraspecies specific, such as *psmα* gene, which downregulates MVs production when deleted in *S. aureus* [10]

EVs are known to package a wide array of cellular compounds such as proteins, lipids, nucleic acids including both DNA (chromosomal, plasmid, or phage origin) and RNA (including mRNA, rRNA, sRNA, and tRNA) [10,12], and other components which are not only heterogeneous with respect to their molecular species [3], but also to their concentration [32,40]. The origin of the EVs, the strain, the biogenesis, and the surrounding conditions can all influence EVs composition, for example, even though EVs from Gram-positive bacteria package similar cargo types to those Gram-negative bacteria, they lack lipopolysaccharides and periplasmic components [30]. In addition, if parent bacteria are pathogenic, they can secrete EVs containing virulence factors (toxins, adhesins and enzymes) [41,42]. In this context, it is important to highlight the term "vesiduction," which refers to the process by which vesicles carrying DNA are released from a donor bacterium, transported to a recipient bacterium, and transfer their genetic material into the recipient's cytoplasm via vesicle fusion with the cell membrane [43].

Based on multiple studies, these cargos are sorted by selective mechanisms that are yet to be elucidated [10,32]. Existing data outline that the general secretion system [32] and the twin-arginine transport (Tat) pathway, which require the presence of a certain signaling domain, are involved in this sorting process in Gram-positive bacteria [10]. However, since some secreted proteins lack the required signal domain, we cannot exclude the existence of alternative mechanisms. In addition, the budding of EVs from the bacterial membrane creates a region of curvature, and it is possible that curvature-detecting proteins could sense these regions and transport components to the correct location in the EVs [10]. It is important to mention that advances in biochemical techniques and technological advances helped in the study of EVs and their compositions. In fact, the similarities in shape, density, components and other properties among the different EVs types render rapid, effective and standardized isolation techniques essential to differentiate between these various types [44]. Existing isolation methods of EVs consist of ultracentrifugation [32,41,42,45], ultrafiltration, and biochemical characterization including size-exclusion chromatography [44]. Though ultracentrifugation is the most commonly used isolation technique, other methods are becoming more popular with all of them having their advantages and disadvantages regarding purity, cost, time and possible contamination. The continuous advancements in research techniques, including isolation characterization and quantification methods will facilitate our understanding of these nanoscale particles.

At the functional scale, EVs are key players in the lifecycle of bacteria, providing diverse means for bacterial communication, survival and adaptation. The fact that they can utilize a wide range of cellular components allows them to have important roles in both inter- and intra-kingdom interactions [42,44,46]. Secretion of signaling molecules, DNA, RNA and proteins in EVs are increasingly viewed as part of the mechanism evolved to allow bacteria complex behaviors such biofilm formation or quorum sensing [13,47,48]. Horizontal gene transfer also helps drive genetic diversity, allowing bacterial populations to quickly acquire beneficial traits like antibiotic resistance [49,50]. EVs in bacteria are reported to be involved in the delivery of toxic materials and modify host immune responses, facilitating thus their evasion, and aiding pathogenic mechanisms established by these deadly organisms [51]. In addition, they help in nutrient acquisition and biofilm formation; protection against environmental threats; or during interaction with host species induce inflammatory response, disrupt cell membranes [10]. Given the diversity relying in EVs compositions, functions, and their emerging interest in different fields, we summarize in this review the different factors that might influence EVs properties, their functions in various fields, and discuss the challenges that face EVs mediated therapies.

## 2 Factors influencing EVs properties

The production, composition, and functions of EVs vary significantly based on multiple factors. Notably, the biological, chemical, or physical stressors that bacteria face in diverse environments or during host infection play a critical role in influencing EV characteristics. Given the limited literature on the impact of these environmental conditions, in vitro studies have been primarily conducted to evaluate EV production [32].

**Temperature** significantly influences EVs, membrane fluidity, their protein expression and their virulence factor packaging [52]. Studies suggest that high temperatures may, enhance EV production, through the increase of membrane fluidity whereas low temperatures can reduce fluidity, leading to fewer vesicles. It is likely that temperature alters membrane fluidity, affecting its curvature and the vesiculation process which was found to be increased with increasing temperatures [52,53]. Likewise, temperature shifts induce changes in bacterial protein folding and stress responses. An example of the effect of temperature on the protein content of *S. aureus* EVs is the observed decrease in protein translation or half-life at higher temperatures. It is important to note that while the localization and functional categorization of proteins remained consistent across different temperatures, proteins associated with energy metabolism, protein synthesis, and virulence factors were predominantly observed at higher temperatures. This indicates that temperature not only impacts the composition but also the function of EVs. For example, if we compare EVs at 40 °C and 34 °C, we notice that for the higher temperature, the cargo is mainly made of virulence factors, while for the lower one, cargo was more diverse. The increased presence of virulence factors at 40 °C suggests that EVs enhance their resistance to the immune response during infection. Conversely, the decreased number of virulence factors at 34 °C could be associated with the colonization phase of *S. aureus*. The effect of temperature on virulence factors packaging was also seen in *Vibrio cholerae* and *Pseudomonas aeruginosa*. Further studies are still necessary to comprehensively understand the functional shifts in EVs at varying temperatures [12].

Another tested stressor is the **osmotic stress** which affects membrane tension which can either promote or inhibit vesicle formation [54]. The significant reduction in EV protein yield is attributed to the thickening of the bacterial cell wall under high osmotic conditions, which hinders EV secretion as shown in *S. aureus* [32]. In other cases, bacteria significantly raises its vesiculation in response to high osmotic pressure as for OMV shedding *P. putida* [54]. For instance, under osmotic stress, EVs might serve as a mechanism for exporting toxic compounds or accumulating molecules that help cells maintain osmotic balance, protecting the cell from damage due to pressure changes, this is known as EV-mediated stress relief [54].

Temperature and osmotic stress are not the only factors that may influence EVs properties, as oxidative stress encountered when reactive oxygen species are produced because of an incomplete depletion of oxygen, has also been described in this context. It triggers the production of EVs enriched in stress-induced molecules, which may signal other cells to induce defense responses or modulate host immune responses. To test this claim, *S aureus* was grown in subinhibitory amounts of ciprofloxacin, an antibiotic known to amplify oxidative stress in bacterial cells. An increase in EVs production in cultures containing ciprofloxacin was shown. One possible explanation could be that bacteria have developed adaptive survival mechanisms in oxidative stress [32].

Understanding how these factors modulate vesicle dynamics provides insights into bacterial survival strategies, their role in pathogenesis, and potential applications in biotechnology or medicine, such as using EVs for vaccines or drug delivery. The continuous advancements in research techniques, including isolation characterization and quantification methods will facilitate our understanding of these nanoscale particles.

## 3 EVs emerging role in various fields

a) EVs pathogenicity and role in immune-response modulation

EVs have emerged as pivotal players in the pathogenesis of various bacterial infections. They help in cell entry, immune system evasion then pathogenesis and tissue damage. Some EVs can fuse with or be taken up by host cells through endocytosis, delivering their cargo directly into the cytoplasm and facilitating infection. For example, *Listeria monocytogenes* EVs assist the bacteria in invading host cells. Additionally, the toxins and enzymes carried by EVs can degrade host tissue barriers, promoting bacterial dissemination and tissue invasion [55]. Additionally, after their secretion by bacteria, these nanoscale, membrane-bound structures facilitate the transfer of virulence factors, toxins, siderophores, adhesins, and signaling molecules to host cells [56]. By delivering pathogenic components directly into host cells, EVs can modulate immune responses, disrupt cellular processes, and enhance bacterial adhesion and invasion [57]. Consequently, EVs are instrumental in manipulating host cell responses, promoting bacterial survival and proliferation within the host. Toxins released by EVs include lipopolysaccharides (LPS), hemolysins, and proteases; to illustrate, *Vibrio cholerae* releases cholera toxin within EVs, facilitating its delivery to intestinal epithelial cells and contributing to diarrheal disease. Additionally, pathogenic bacteria such as *P. aeruginosa* release EVs containing degradative enzymes like proteases and lipases, which promote tissue damage, nutrient acquisition, and host tissue invasion [15,55].

EVs interact largely also with Toll-like receptors (TLRs), which are a class of pattern recognition receptors (PRRs) found on the surface of various immune cells, such as dendritic cells, macrophages, and neutrophils [58]. EVs and TLRs are closely interconnected in the regulation of immune responses. EVs can carry various pathogen-associated molecular patterns (PAMPs) such as lipopolysaccharides (LPS), viral RNA, and bacterial DNA, which are recognized by TLRs on immune cells, thereby triggering an immune response, and providing protection against infection and invasion, while also enhancing tissue repair pathways [59]. Additionally, EVs may contain damage-associated molecular patterns (DAMPs) from stressed or dying cells, further activating TLRs. This interaction leads to the activation of immune cells like dendritic cells and macrophages, inducing cytokine production, antigen presentation, and inflammation [60]. In disease contexts, such as infections or cancer, EVs can modulate immune responses by either promoting or suppressing TLR signaling, depending on their origin and contents. For instance, tumor-derived EVs may either evade immune detection by dampening TLR signaling or activate TLRs to initiate an anti-tumor immune response. Moreover, EVs are being explored as potential vehicles for delivering TLR agonists or antagonists in therapeutic applications, enhancing vaccine efficacy or modulating excessive inflammation in autoimmune diseases [[61], [62], [63]].

The manipulation of host's immune defenses allows for a persistent infection. EVs can also act as decoys, absorbing antibodies and antimicrobial peptides that would otherwise target the bacterial cell surface, enabling bacteria to avoid direct immune attacks. As an example, EVs from pathogens like *Helicobacter pylori* and *Neisseria meningitidis* contain immune-modulatory factors that inhibit immune cell activation, downregulate inflammatory responses, or trigger apoptosis in immune cells, further helping bacteria evade clearance [64,65].

**Fig. 2:**
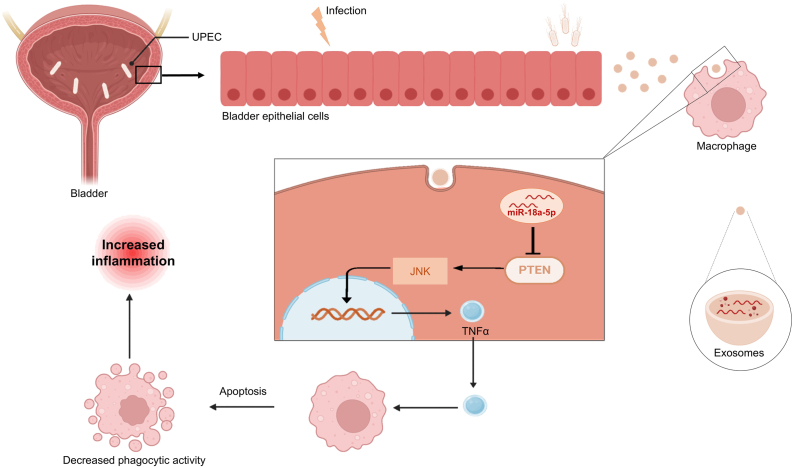
**Schematic illustration depicting exosomes originating from bladder epithelial cells following Uropathogenic *Escherichia coli* (UPEC) infection and their detrimental effect on macrophage function.** These exosomes, once released, are taken up by macrophages, leading to the activation of the JNK signaling pathway and the secretion of TNFα. This occurs through the delivery of miR-18a-5p, which suppresses PTEN expression, resulting in macrophage apoptosis and reduced phagocytic activity.

This sophisticated mechanism of intercellular communication underscores the significant role of EVs in the development and progression of bacterial diseases, highlighting their potential as targets for therapeutic intervention and diagnostic markers in infectious diseases such as, urinary tract infections (UTIs), caused by Gram-negative, Gram-positive bacteria, and fungi [66]. For example, during UTIs caused by Uropathogenic *Escherichia coli* (UPEC), exosomes function as critical communication mediators between bladder epithelial cells and macrophages. UPEC infections stimulate the release of exosomes from bladder epithelial cells, which are subsequently internalized by macrophages. This process results in an excessive production of pro-inflammatory cytokines, particularly tumor necrosis factor alpha (TNFα). The heightened TNFα production significantly exacerbates tissue damage. Accordingly, targeting the inhibition of exosome release or TNFα activity presents a promising therapeutic strategy to mitigate the severity of UPEC-induced UTIs (Fig. 2) [67]. During Gram-negative bacterial infections, OMVs play a vital role in delivering lipopolysaccharide (LPS) into the cytosol, thereby activating caspase-11-mediated immune responses in host. As such, OMVs are indispensable for inducing caspase-11-dependent cell death and cytokine production. Bacterial strains with reduced vesiculation, which produce fewer OMVs, provoke weaker immune responses compared to wild-type strains. Upon endocytosis by macrophages, OMVs release LPS from early endosomes into the cytosol, ensuring the delivery of intact LPS. This elucidation of OMVs as crucial vehicles for LPS transport and immune activation holds significant implications for vaccine development and the broader understanding of host-pathogen interactions [68]. Subsequently, EVs are involved in regulating immune processes and are implicated in allergic, autoimmune, and metabolic diseases.

b) Role of EVs in Inducing Antimicrobial Resistance and Effect of Antibiotics on EVs

EVs play a significant role in the spread of antimicrobial resistance (AMR) among bacterial populations, enhancing thus the likelihood of resistance transmission, impacting both clinical and environmental settings and complicating the treatment of bacterial infections [69]. Gene dissemination, is one of the key actions played by EVs, which can carry and transport genetic material, including plasmids and antibiotic resistance genes, between bacteria. This horizontal gene transfer also known as vesiduction facilitates the rapid spread of AMR traits within and between bacterial species, enabling previously susceptible bacteria to acquire resistance. Vesicles containing resistance genes DNA are released from the surface of the donor bacterium, transported to the recipient bacterium, and fuse with its cell membrane to deliver their contents into the cytoplasm [70]. In details, the vesiduction process through which a susceptible bacteria could become resistant to one or more antibiotic families occur through several steps: (i) Vesicles containing DNA encoding for resistance genes are expelled from the donor resistant bacterium, (ii) this vesicle migrates to reach the recipient susceptible bacterium, (iii) vesicles attach to recipient bacterium's surface, (iv) the DNA encoding resistance genes is then transferred to the recipient bacterium's cytoplasm leading to (v) the spread of antibiotic resistance [70]. One major example of resistance genes carried by are β-lactamases. Carrying these enzymes induce resistance spread against critical antibiotics like β-lactams [69]. For instance, *Acinetobacter baumannii*, releases OMVs carrying *bla*_OXA-58_, *bla*_OXA-23_ and *bla*_NDM-1_ genes encoding oxacillinases of β-lactamases family which represents a significant challenge in combatting AMR. This enables bacteria to hydrolyze carbapenems and thus constitutes a line of defense for *A. baumannii* [69,71]. Moreover, OMVs originating from *Stenotrophomonas maltophilia* and other resistant bacterial strains, such as *E. coli*, *Moraxella catarrhalis*, and *Bacteroides fragilis*, transport enzymes like β-lactamases [72]. The presence of these enzymes highlights the protective function of vesicles under stress conditions. By producing and secreting these enzymes within vesicles, bacteria not only protect themselves from β-lactam antibiotics but also extend this resistance to other bacterial species, such as *Pseudomonas aeruginosa* and *Burkholderia cenocepacia*. This mechanism underscores the critical role of vesicles in mediating interspecies resistance and enhancing bacterial survival under antibiotic pressure [73]. Other studies showed that, *Haemophilus influenzae* synthesizes β-lactamase and encapsulates it within vesicles. These vesicles, laden with β-lactamase from *H. influenzae*, may be secreted by bacteria, potentially including Group A *Streptococcus*, to shield themselves from the antimicrobial effects of amoxicillin. This strategic packaging and release mechanism highlights a sophisticated means of interbacterial cooperation and resistance dissemination [74]. In the same context, EVs play a crucial role in the horizontal transfer of antibiotic resistance genes among extended spectrum β-lactamases ESBL-producing *E. coli* strains. The transfer frequency of genes like *bla*_CTX-M-55_ increases under antibiotic pressure, suggesting that EVs significantly contribute to the spread of antibiotic resistance, particularly in high-antibiotic environments [75]. To elaborate, EVs from *Streptococcus pneumoniae* facilitate horizontal gene transfer by carrying DNA on their external surfaces, which can be taken up by pneumococcal cells. These findings highlight EVs' role in spreading antibiotic resistance genes, providing new insights into bacterial evolution and antibiotic resistance [76,77].

Not only do EVs carry resistance genes, but they also protect this genetic material by encapsulating the genes and their associated regulatory elements, shielding them from degradation in the extracellular environment. This enhanced stability increases the chances that recipient bacteria will successfully incorporate the genetic elements, thereby promoting the spread of AMR [78]. Conversely, exposure of bacteria to antibiotics also affects the production of EVs, as the case of *Geobacter sulfurreducens* exposure to sub-minimal inhibitory concentrations of antibiotics which affected the production and redox activity of OMVs. Antibiotics like ampicillin and ciprofloxacin induce different OMV biogenesis mechanisms, with ciprofloxacin-induced OMVs containing more redox-active molecules. These antibiotic-induced OMVs also exhibit increased DNA content, suggesting a stimulation of OIMVs (outer inner membrane vesicles) biogenesis via explosive cell lysis [79]. A study on *E. coli* strain K1 RS218 reveals that β-lactam antibiotics significantly increase BEV production, especially at clinical concentrations, whereas aminoglycosides do not. This suggests that high doses of β-lactam antibiotics used in sepsis treatment may exacerbate inflammation due to increased BEV release, highlighting the need for optimized antibiotic dosing to balance effective infection control with minimal inflammatory side effects [80]. Interestingly, EVs derived from airway epithelial cells transfer small RNA fragments to *P. aeruginosa*, increasing its sensitivity to fluoroquinolones. These EVs reduce the protein levels of the MexHI-OpmD efflux pump, with significant reductions observed in MexH and OpmD protein levels due to an 18S rRNA fragment, suggesting that, eukaryotic rRNA fragments delivered via EVs can regulate prokaryotic gene expression and phenotype, presenting potential therapeutic applications against antibiotic-resistant infections [81].

All cited studies prove that the role of EVs in the spread of antimicrobial resistance is multifaceted, encompassing gene transfer, biofilm dynamics, immune evasion, and environmental dissemination. Understanding the mechanisms by which EVs contribute to AMR spread is crucial for developing effective strategies to combat antibiotic resistance and improve infection control measures. Targeting bEV production or function may offer novel approaches to mitigate the spread of AMR in clinical and environmental contexts.

c) Antimicrobial and Antibiofilm Properties of OMVs

Regarding AMR, it presents a significant obstacle in antimicrobial therapy, especially in biofilms. Biofilms consist of intricate communities of microorganisms, such as bacteria, encased in a self-produced extracellular matrix that shields them from antibiotics and the host's immune defenses [82]. Biofilm resistance to antibiotics far exceeds that of planktonic (free-floating) bacteria. Biofilms constitute a survival strategy for microorganisms, allowing them to withstand harsh conditions by resisting to external stressors, such as antibiotics, or the host immune system, leading to their virulence [83]. Biofilm modulation is composed of multiple steps including attachment, adaptation, exponential growth, maturation, stationary growth, and dispersion, which are tightly regulated by active cell-to-cell communication via signaling mediators, such as EVs [84]. EVs play critical roles in biofilm formation, structural integrity, matrix composition and function, contributing to resilience, communication, and interaction within these communities. Acting as carriers of proteins, lipids, nucleic acids, and signaling molecules, EVs facilitate intercellular communication, strengthening the biofilm structure and enabling bacteria to exchange genetic material, virulence factors, and other bioactive compounds [85]. This communication helps coordinate biofilm development, strengthens the biofilm structure, maintains the protective extracellular environment for bacteria and helps them adapt to environmental changes [86,87]. Regarding biofilm structure, EVs enhance biofilm stability and cohesion by cross-linking various matrix components, such as proteins, polysaccharides, and DNA, within the biofilm. The cross link is facilitated by the bEV's membrane structure that it similar to that of the originating bacteria making the bEV able to mimic bacterial interactions with its surrounding environment, notably the ECM. This cross-linking creates a dense, interconnected network that holds the biofilm together, reinforcing its structure and making it more robust. As a result, the biofilm becomes more resistant to physical disturbances like shear forces or environmental changes. Additionally, the increased structural integrity provides a protective barrier against antimicrobial treatments, making it harder for antibiotics to penetrate and reach the bacteria embedded within the biofilm. This enhanced resistance contributes to the persistence of biofilm-associated infections and bacterial survival mediated by their EVs [13,87].

Several studies have explored the role of EVs in biofilm formation or inhibition, and the modulation of virulence. EVs could contribute to the biofilm's resistance to antibiotics, by mediating the transfer of antibiotic resistance genes between bacteria, contributing further to antibiotic resistance [88]. Moreover, EVs carry quorum sensing molecules that allow bacteria within the biofilm to communicate and coordinate activities, such as virulence factor production, biofilm maturation, and resistance mechanisms. This communication is critical for the biofilm's ability to adapt to environmental challenges. The delivery of quorum sensing signals via EVs helps synchronize the behavior of bacterial populations, promoting the growth and development of the biofilm in response to external stimuli [89,90].

While EVs promote biofilm formation and virulence, they can also prevent early-stage biofilm formation, and even reduce virulence under certain conditions. For instance*, Lacticaseibacillus casei* vesicles affects the early stages of *Salmonella Enteritidis* biofilm formation and prevents attachment of bacteria to polystyrene surfaces, through the peptidoglycan hydrolase released in their vesicles [91]. Likewise, *Pseudomonas aeruginosa* produces OMVs, that exhibit antibiofilm activity, through endogenous proteases [92,93]. Other examples of biofilm inhibition include OMVs produced by *Burkholderia thailandensis,* which inhibits biofilm biomass and integrity in *Streptococcus mutans* [94]. However, some EVs enhance biofilm formation, as for Aeromonas strain OMVs, which enhances biofilm formation in a dose dependent manner [95], *Staphylococcus aureus* and *Streptococcus mutans* which increases biofilm formation [96,97]. This duality of roles suggests potential therapeutic uses of EVs in inhibiting biofilm formation and as a result reducing bacterial virulence.

Other studies exploring the roles of EVs secreted by *P. aeruginosa* biofilms in controlling biofilm growth, found that EVs secreted during the exponential growth phase promote biofilm growth, while EVs secreted during the death/survival phase can potentially reduce or inhibit biofilm growth, reinforcing their implication in cell-to-cell communication. Moreover, the function of EVs correlates with the conditions of the biofilm growth stage, thus, growth phase-EVs promote biofilm growth, while death phase-EVs inhibit biofilm formation and growth. As a result, researchers believe that death phase EVs can be used to treat *P. aeruginosa* biofilms at the early stage of biofilm growth. This inhibition of biofilm growth by death phase EVs is associated with reactive oxygen species production (ROS). This discovery could provide an alternative for developing new therapies against drug-resistant bacterial infections. It is crucial to mention that, in some cases, EVs derived from non-bacterial sources could inhibit bacterial biofilm formation, as the case of the fungi *C. albicans* which produces EVs that negatively impact the adherence and biofilm formation of the bacterial pathogen *Klebsiella pneumoniae*, suggesting a potential antagonistic action of fungal EVs against bacteria [98].

d) EVs therapeutic applications

EVs, including OMVs from Gram-negative bacteria and MVs from Gram-positive bacteria, represent a rapidly advancing frontier in therapeutic research [99]. As mentioned before, recent advancements underscore their potential to modulate immune responses and deliver therapeutic payloads with high precision [100]. As our understanding of EVs deepens, their medical applications could revolutionize disease treatment and management, offering a groundbreaking convergence of microbiology and therapeutic innovation EVs biocompatibility and safety, combined with the ability to be engineered for specific medical purposes, position EVs as a versatile and powerful tool in modern medicine, showing thus a significant promise for clinical applications including cancer therapy, and infection control [99,101].

Recently, EVs have garnered significant interest as a potential solution to antibiotic resistance. They exhibit potent bactericidal capabilities, making them effective against a variety of pathogens and they offer a promising platform for antibiotic delivery, enhancing the efficacy and specificity of treatments [102]. As research continues, the multifaceted applications of EVs could revolutionize the fight against antibiotic resistance and transform infectious disease management [102]. Improving the in vivo activity of antibiotics to eradicate bacterial infections is crucial, especially that, sometimes, free antibiotics are not able to accumulate effectively in the infection site, due to a poor utilization rate, as for fluoroquinolones, which can cause several adverse reactions in tendons, muscles, joints and nervous system, leading to toxicity in some cases [103]. Using vesicles as antibiotic delivery systems may be a solution to better target the infection sites more specifically [103]. In fact, promising results were obtained after loading ciprofloxacin in vesicles to treat *Shigella* infected models [104]. As mentioned before, encapsulating ciprofloxacin or any other antibiotic could lead to a more targeted and efficient treatment, enhancement of pharmacokinetics, and reduction of side effects. antibiotic delivery systems face numerous challenges, including the difficulty of penetrating bacterial biofilms, the need for targeted delivery to avoid off-target effects, and the stability of the antibiotic within the delivery vehicle. The unique capacity of EVs to deliver molecules to the envelope of Gram-negative bacteria suggests that they may act as natural antibiotic delivery vehicles, potentially overcoming the limitations of existing methods. Their stability and ability to protect their luminal contents from enzymatic degradation further enhance their suitability as antibiotic delivery systems. Moreover, EVs play a significant role in modulating host-pathogen interactions which underscores the potential of EVs not only in therapeutic applications but also in understanding bacterial pathogenesis and developing novel antimicrobial strategies. By leveraging the inherent properties of EVs, researchers aim to create more effective and targeted treatments, addressing the critical issue of antibiotic resistance and improving infection control [88,101].

The role of EVs in cancer treatment is of particular interest, especially in the context of gastric cancer associated with *Helicobacter pylori* infection. To prevent this type of cancer, a combination of standard antibiotic therapy with probiotics and postbiotics has shown significant efficacy in managing *H. pylori* infections. The modulation of *H. pylori*-induced inflammation can be achieved by downregulating the mRNA expression of pro-inflammatory cytokines such as IL-1β, IL-6, IL-8, and TNF-α, while upregulating the expression of anti-inflammatory cytokines like IL-10 and TGF-β in AGS cells. Notably, EVs derived from *Lactobacillus crispatus* have been found to decrease IL-8 production, suggesting for the first time that EVs released by the *L. crispatus* strain could be recommended as potential therapeutic agents against H. pylori-triggered inflammation, which can lead to gastric cancer. This innovative approach highlights the potential of EVs not only in delivering targeted therapies but also in modulating immune responses to prevent cancer development. By integrating EVs into treatment protocols, we can enhance the effectiveness of current therapies, reduce inflammation, and potentially inhibit the progression of infection-related cancers. The versatility and precision of EVs represent a promising frontier in the fight against cancer and other diseases influenced by microbial infections [105,106].

EVs have shown substantial promise in cancer therapy, particularly for drug delivery [101]. A notable study by Kuerban et al. (2020) investigated EVs derived from *Klebsiella pneumoniae*, focusing on the development of doxorubicin-loaded outer membrane vesicles (DOX-OMVs) [107]. Doxorubicin (DOX) is a widely used chemotherapy drug known for its efficacy in treating various cancers. The study demonstrated that these DOX-OMVs could effectively deliver doxorubicin to lung cancer cells and significantly inhibit tumor growth in mouse models. Moreover, the research indicated that these OMVs could recruit macrophages within the tumor microenvironment (TME), thereby enhancing the in vivo cytotoxicity of doxorubicin. This innovative approach facilitates the targeted delivery of chemotherapeutic drugs, minimizing off-target toxicity, triggering appropriate immune responses, and inhibiting cancer progression [107]. The ability of EVs to modulate the TME and enhance drug efficacy underscores their potential as a transformative tool in cancer therapy. By leveraging the unique properties of EVs, researchers are opening new avenues for precision medicine, where treatments can be tailored to target specific cancer cells while preserving healthy tissues. This improves therapeutic outcomes and reduces the adverse effects associated with conventional chemotherapy. As our understanding of EVs continues to expand, their integration into clinical practice could revolutionize the landscape of cancer treatment, offering hope for more effective and personalized therapies.

Furthermore, OMVs have been engineered to target specific cancer cells using *E. coli*-derived OMVs. A transdermal nanoplatform for melanoma therapy was developed in mice by modifying *E. coli*-derived OMVs with an αvβ3 integrin targeting ligand and indocyanine green [108]. When applied topically to the melanoma site, these OMVs could penetrate the stratum corneum and target melanoma cells with high specificity through the interaction between the αvβ3 integrin and the ligand on the cell surface. Upon photothermal irradiation with near-infrared light, indocyanine green dissociates from the OMVs, inducing both peroxidase-antiperoxidase activity and hyperthermia, leading to necrosis in tumor spheroids [108]. This photothermal effect also deforms the OMVs, causing the release of tumor necrosis factor-related apoptosis-inducing ligand (TRAIL), which then binds to death receptors on the cancer cell surface, activating apoptosis. This combined photothermal and OMV treatment significantly increased antiproliferation rates by 20–50 % and decreased invasion capacity by 20–76 % in melanoma cell lines [108]. Additionally, it prevented tumor relapse and metastasis by interfering with specific genes and proteins. This approach offers notable advantages in terms of safety and biocompatibility, highlighting the therapeutic potential of engineered EVs in cancer treatment [109].

The anti-cancer effects of OMVs derived from *E. coli* W3110 in colon cancer. aggregates at tumor sites, inducing a long-term antitumor immune response that resulted in tumor regression. Additionally, they promoted the production of the anti-cancer cytokine CXCL10 and interferon-γ. The study proposed that these OMVs could be used as immunotherapeutic agents for several types of cancer without apparent side effects [110,111]. These advancements underscore the versatility and potential of engineered EVs in oncology, by enabling precise targeting and delivery of therapeutic agents, where the integration of EVs into clinical cancer therapy could revolutionize the field, offering new hope for effective and personalized treatment strategies.

Overall, EVs hold promising potential in the therapeutic field. By serving as natural carriers of bioactive molecules, including antigens and signaling compounds, they can be engineered for targeted drug delivery, and immunomodulation. Additionally, understanding their role in pathogenesis offers insights for novel antimicrobial strategies, such as disrupting bEV production or blocking their interactions with host cells to combat infections and limit the spread of antimicrobial resistance. Harnessing EVs in therapeutics opens new avenues for precise and effective treatments in infectious disease management.

e) EVs role in synergy between bacterial species

EVs derived from *S. aureus* play a key role in mediating the interaction between *S. aureus* and *P. aeruginosa* [112]. *S. aureus* derived EVs (SaEVs) can fuse with the membrane of *P. aeruginosa*, thus, a direct interaction between *S. aureus* and *P. aeruginosa* was confirmed, where SaEVs enhanced Lipopolysaccharide (LPS) production in *P. aeruginosa*, increasing their pathogenicity. This increase in LPS production can potentially increase the bacterial ability to cause disease and evade the host immune system. Similarly, enhanced biofilm formation was denoted, ensuring the primary role of SaEVs is to modulate pathogenic traits in *P. aeruginosa*, thus their synergistic correlation. Four unique proteins from SaEVs, not found in *P. aeruginosa*, were detected in the fused bacteria, confirming that the proteins are transferred from SaEVs to *P. aeruginosa*, potentially endowing the latter with new capabilities. Therefore, SaEVs promote synergism between *S. aureus* and *P. aeruginosa*, as SaEVs enhance the pathogenicity of *P. aeruginosa* by promoting LPS biosynthesis, enhancing biofilm formation, increasing the invasion of host epithelial cells, and reducing uptake by immune cells, specifically macrophages. This synergy complicates infections and increases their severity especially in poly-microbial infections where both bacteria are present [112]. Furthermore, in the human gut microbiome, *Bacteroides thetaiotaomicron* and *Faecalibacterium prausnitzii* exhibit a synergistic interaction facilitated by EVs. In fact, *B. thetaiotaomicron* releases EVs containing enzymes that break down complex polysaccharides into simpler sugars, which are then utilized by *F. prausnitzii* for growth. In return, *F. prausnitzii* produces butyrate, a beneficial short-chain fatty acid. The EVs also contain signaling molecules that enhance butyrate production, creating a mutualistic relationship that promotes gut health and stability [113].

The synergy between bacterial species, facilitated by EVs, plays a crucial role in shaping microbial communities by enabling interspecies communication through the transfer of signaling molecules, genetic material, and metabolites, promoting cooperative behaviors such as biofilm formation, resource sharing, and enhanced resistance to environmental stressors and antibiotics. This interaction can enhance the virulence and survival of bacterial populations, making infections more difficult to treat. Understanding this synergy is essential for developing strategies to disrupt harmful bacterial collaborations and improve infection control.

f) Role of EVs in inducing plant infection

EVs are fundamental mediators of plant-pathogen interactions and environmental adaptation. Plant immune responses can be triggered by EVs, emphasizing on the strategic role of EVs in plant infection. The adaptive functions of EVs enables the pathogen to thrive in the plant environment, by demonstrating its roles in antibiotic defence and iron acquisition. EVs can initiate immune responses in plants mediated by bacterial flagellin, a component recognized by the plant immune system [114]. This shows that EVs can play a role in the early stages of plant-pathogen interaction by modulating host immunity. EVs contain virulence proteins that could potentially suppress plant immune responses, aside from initiating an immune response, EVs could potentially help plants adapt to environmental factors. It was further revealed that bacterial survival in the presence of plant-derived antimicrobial compounds was enhanced by the presence of β-lactamase in EVs, suggesting a mechanism for antibiotic resistance [115]. Additionally, under iron-limiting conditions, such as those encountered in the plant apoplast, it is believed that EVs contribute to iron acquisition, due to the enrichment of siderophore transport proteins in EVs. This suggests that EVs participate in environmental adaptation in plants. Therefore, EVs have multifaceted roles. Participation in plant infection, and environmental adaptation.

Through the delivery of virulence factors and modulation of host immune responses, EVs play a pivotal role in the infection process. Furthermore, their involvement in antibiotic resistance and iron acquisition underscores the adaptive benefits of EVs in supporting bacterial survival and proliferation within the plant host, which sheds light on the intricate interplay between bacterial pathogens and their plant hosts, offering potential targets for the development of novel plant protection approaches and strategies to manage bacterial diseases in agriculture.

g) Vaccination and preventive medicine

Given that EVs carry pathogen-associated molecular patterns that activate innate immune receptors and contain antigens that provoke B and T cell responses, they effectively stimulate both innate and adaptive immune responses. Consequently, EVs emerged as promising candidates for vaccine development due to their unique properties and abilities to elicit strong immune responses [56]. EVs can encapsulate and deliver various antigens, including proteins, lipopolysaccharides, and toxins, which are critical for stimulating an immune response. Their ability to present these antigens in a native conformation enhances their immunogenicity (Table 3) [116].

**Table 3:** Key properties and functions of extracellular vesicles in vaccine development.

EV feature	Role in vaccine development	Applications
Immune activation	Stimulates both innate and adaptive immune responses through pathogen associated molecular patterns and antigen presentation.	Enhances immunity against pathogens and cancer cells.
Antigen encapsulation	Encases and delivers antigens like proteins, lipopolysaccharides, and toxins in their native form, improving their immunogenicity.	Enables antigen-specific immune responses critical for effective vaccination.
Versatility in applications	Targets infectious diseases and cancer by utilizing EVs' antigen presentation capabilities to provoke immune responses.	Includes cancer immunotherapy and combating resistant bacteria such as *Enterococcus faecium.*
Targeted delivery mechanism	The nanoscale size and biocompatibility of EVs allow for precise delivery of antigens to immune cells enhancing uptake and efficacy.	Improves targeting of immune cells leading to more efficient immune responses.
Stabilization of components	Protects delicate vaccine components ensuring stability during storage and transport even under strict cold chain conditions.	Critical for vaccines requiring temperature-controlled logistics such as mRNA vaccines.

EVs can serve as vaccines not only for infectious diseases, but also for cancer, by presenting cancer antigens within the structure of the extracellular vesicles, EVs had a role in stimulating the immune system to recognize and attack cancer cells, thus, shedding light on the natural adjuvant properties of EVs to enhance the body's immune response against cancer [22].

Besides, EVs derived from *Enterococci* serve as potential vaccine candidates [117]. *Enterococcus faecium*, including vancomycin resistant *Enterococci* (VRE) notorious for causing nosocomial infections, especially in immunocompromised patients, poses a threat on the healthcare system [118]. To overcome drug resistance and combat these infections, *Enterococci* derived vaccines could prevent infections and act as a promising alternative to antibiotics [117]. Most specifically, six proteins associated with extracellular membrane vesicles produced by *E. feacium*, are the potential vaccine candidates [117]. This study highlights that these naturally occurring *E. feacium* MVs can be utilized as a multi-antigen platform to induce protective immune responses against enterococcal infections [117].

It's crucial to mention that the nanoscale size and natural biocompatibility of EVs allow them to serve as targeted delivery systems for vaccines where they can facilitate the transport of vaccine components to specific immune cells, enhancing uptake and efficacy. In addition, they can stabilize sensitive vaccine components, protecting them from degradation during storage and transport, which is particularly beneficial for vaccines requiring strict cold chain logistics [119,120].

Taken together, EVs represent a promising avenue for vaccine development due to their ability to enhance immunogenicity, stability, and targeted delivery of antigens. Their natural properties as carriers and adjuvants can lead to the development of innovative and effective vaccines that can improve immune responses against a variety of pathogens. As research in this area continues to evolve, EVs may play a crucial role in shaping the future of vaccination strategies (Table 3).

h) EV role as diagnostic tools

Recently, EVs have emerged as powerful tools in both research and diagnostics due to their unique properties and functions. For instance, they can be used as potential, disease biomarkers, that helps in a targeted and rapid diagnosis through non-invasive sampling from body fluids such as blood, urine, or saliva [101]. Loaded with proteins and miRNAs, they serve as potential biomarkers for diagnosing and predicting the progression of various diseases, including distinct types of cancer [101], such as lung cancer. For example, measuring epidermal growth factor (EGFR) receptors on exosomes could be valuable for in vitro diagnosis, with exosomal EGFR being a potential biomarker for characterizing lung cancer [121]. Likewise, serum exosomal miR-378 has strong potential as a promising non-invasive biomarker for screening and monitoring non-small-cell lung cancer [122]. Alternatively, acute ischemic stroke (AIS) can also be detected through the cargo of serum exosomes. A high correlation between the protein SIRT2, was found in AIS patients suggesting that serum exosomes carrying this protein serve as a characteristic biomarker of AIS. Therefore, SIRT2 concentration in serum exosomes can be used to predict the diagnosis of AIS [123]. Another interesting example is the use of urinary exosomal microRNA as biomarkers to detect acute rejection in kidney transplant patients [124,125].

To conclude, EVs present promising opportunities for improving diagnostic methods, particularly in point-of-care settings, where rapid and accurate identification of bacterial infections is crucial.

i) bEV health promoting effects

**Fig. 3:**
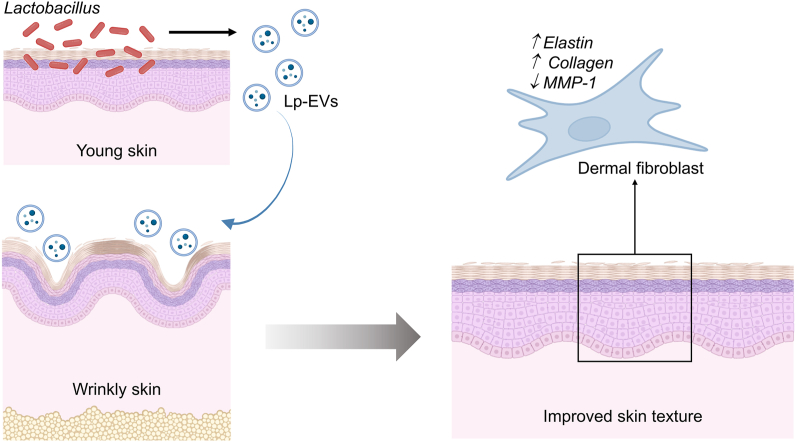
**The anti-aging effects of extracellular vesicles derived from Lactobacillus plantarum.***L. plantarum*-derived extracellular vesicles (LpEVs) isolated from the skin of women in their 20s enhance fibroblast proliferation, upregulate collagen while downregulating MMP-1, reduce wrinkle formation, and increase skin moisture content, suggesting potential benefits in preventing skin aging.

EVs derived from prokaryotes, and eukaryotes were highly studied for their regenerative and therapeutic potential, especially in skin regeneration and wound healing. For example, mesenchymal stem cells-derived EVs (MSC-EVs) enhance angiogenesis by delivering growth factors like vascular endothelial growth factor to injury sites, accelerating tissue repair and promoting sustained healing. They also stimulate the proliferation of epithelial cells and fibroblasts, essential for wound closure and extracellular matrix remodeling, thereby aiding in the restoration of normal skin architecture. Additionally, MSC-EVs support skin health and hair growth, showing promise in treating conditions like alopecia [[126], [127], [128]]. EVs were also able to offer similar benefits. For example, EVs derived from *Leuconostoc holzapfelii*, a bacterium found on the human scalp, have shown promising results in promoting hair growth. In studies involving human follicle dermal papilla cells, these EVs stimulated cell division, migration, and proliferation, displaying hair-inductive activity. Moreover, EVs such as those derived from *Lactobacillus plantarum* (LpEVs), isolated from the skin of young Korean women, have demonstrated significant anti-aging and anti-pigmentation effects (Fig. 3) [129]. LpEVs were found to modulate extracellular matrix-related genes, increasing the expression of pro-collagen type I (COL1A1) and decreasing the expression of matrix metalloproteinase-1 (MMP-1). Additionally, LpEVs exhibited potent elastase inhibitory activity, suggesting their potential to prevent elastin degradation and maintain skin elasticity. Clinical trials revealed that participants treated with LpEVs experienced reduced wrinkle formation and pigmentation, highlighting their potential as anti-aging agents [129]. While more research is necessary, the emerging evidence suggests that EVs, both MSC-derived and bacterial, hold significant promise for addressing beauty-related concerns such as skin aging, thinning hair, and alopecia. These findings open exciting possibilities for the development of innovative skincare products and treatments aimed at enhancing skin health and appearance.

## 4 Challenges facing EV-mediated therapies

As mentioned above, EVs, including exosomes and other microvesicles, have gathered significant attention as potential therapeutic vehicles due to their natural biocompatibility, ability to carry diverse molecular cargo, and inherent targeting capabilities. Despite the vast therapeutic applications of ECs, several challenges are associated with EV-mediated therapies and were underscored in several research.

One of the primary challenges in EV-mediated therapies is the production and scalability of these EVs. Meyer et al. (2024) acknowledged the difficulty in producing enough Streptomyces-derived EVs [130], while Zhou et al. (2023) emphasized the labour-intensive process of isolating exosomes from tumor cells [131]. Therefore, producing EVs is challenging due to low yields, and a demanding laborious process.

Furthermore, efficient drug loading and stability of EVs are critical for their therapeutic efficacy. To overcome this issue, Zhou et al. addressed this by creating hybrid exosomes, which combine the stability of liposomes with the targeting ability of exosomes [131]. However, achieving consistent and high drug loading efficiency remains a challenge. The stability of drug-loaded EVs during storage and in physiological conditions is also a concern that needs to be addressed to ensure reliable therapeutic outcomes. While EVs have natural targeting abilities, enhancing their specificity and efficiency remains an obstacle [131]. In this same context, Meyer et al. focused on the necessity of optimizing targeting mechanisms to minimize off-target effects [130].

Although EVs are considered biocompatible, immunogenicity and safety cannot be ignored. The need for thorough evaluation of the immunogenicity and long-term safety of EV-based therapies, due to possible immune responses should not be underscored [130], particularly when derived from bacteria, or when used in repeated doses. While EV-mediated therapies hold great promise for a variety of medical applications, from antimicrobial treatments to targeted cancer therapy, key challenges including production scalability, efficient drug loading, targeted delivery, and ensuring safety and biocompatibility remain to be solved. Addressing these challenges through innovative approaches and rigorous research will be crucial for the successful translation of EV-based therapies into clinical practice.

## 5 Conclusion

**Fig. 4:**
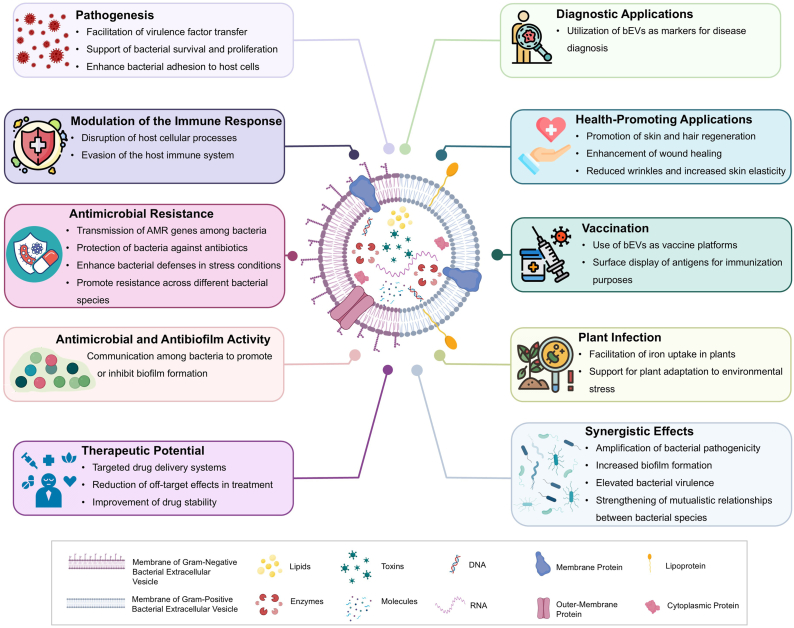
**Extracellular Vesicles Various Applications**. EVs functions involve, but are not limited to, pathogenesis, modulation of the immune response, antimicrobial resistance, antimicrobial and antibiofilm activity, and plant infection. EVS also have a therapeutic potential as they have health-promoting effects, and could be used for vaccination and disease diagnosis. It is important to note that EVs also have synergistic effects thus influencing their physiology, interaction, and interaction with their environment.

To conclude, EVs are involved in various physiological and pathological processes, broadening their applications and emphasizing the benefits of understanding their biology (Fig. 4). As such, they are vital for bacterial survival and adaptation due to their diverse physiological roles. These nanoscale particles are key players in bacterial communication, nutrient acquisition, and defense mechanisms. Physiologically, EVs enable bacteria to communicate with one another by transferring signaling molecules, DNA, RNA, and proteins, which orchestrates collective behaviors like biofilm formation and quorum sensing. They also facilitate horizontal gene transfer, allowing for the rapid spread of advantageous traits such as antibiotic resistance across bacterial populations. Furthermore, pathogenic bacteria use EVs to deliver toxins and modulate host immune responses, enhancing their ability to infect and evade the host's defenses. EVs also assist in nutrient acquisition by carrying enzymes that degrade complex substrates, contributing to the bacteria's survival in nutrient-limited environments. From a diagnostic and therapeutic perspective, EVs hold immense potential due to their unique properties. Their ability to carry pathogen-associated molecular patterns and antigens makes them valuable for developing innovative vaccines and diagnostic tools. By leveraging EVs to present these antigens, researchers can create vaccines that elicit strong immune responses against specific pathogens. Moreover, EVs can be engineered to deliver therapeutic agents or genes directly to targeted cells, offering a novel approach to treating infections.

Despite these promising applications, several challenges face their use in the medical field, including the need for better understanding of EV biology, their efficient isolation and characterization techniques, and overcoming regulatory hurdles. Nevertheless, ongoing research and technological advancements are paving the way for the effective use of EVs in clinical settings. As we continue to explore and refine these applications, EVs can significantly impact diagnostics and therapeutics, offering new strategies for managing and treating a wide range of diseases.

Through a unique framework that incorporates findings from many studies, our review offers a thorough synthesis of EVs' features, highlighting new applications and providing practical insights. Because of this, it is a useful tool for both practitioners and researchers. But it's important to recognize several limitations, like the possible exclusion of research written in languages other than English and the scarcity of reliable data in particular fields, which could restrict the applicability of our findings. Despite these constraints, our review's advantages: its thoroughness of analysis, critical assessment of results, and emphasis on filling knowledge gaps, significantly outweigh its limitations, laying a solid foundation for future research to build upon.

## CRediT authorship contribution statement

**Mariam Rima:** Writing – original draft, Writing – review & editing. **Mariam Dakramanji:** Writing – original draft, Visualization. **Elie El Hayek:** Writing – original draft, Visualization. **Tia El Khoury:** Writing – original draft, Visualization. **Ziad Fajloun:** Project administration. **Mohamad Rima:** Writing – review & editing, Supervision, Conceptualization.

## Data and code availability statement

No new data was generated for the research described in the article.

## Declaration of competing interest

The authors declare that they have no known competing financial interests or personal relationships that could have appeared to influence the work reported in this paper.
